# Neuromuscular training for preventing knee injuries in female team athletes: a meta-analysis

**DOI:** 10.1080/07853890.2025.2581891

**Published:** 2025-11-01

**Authors:** Jinfa Gu, Ruohan Zhang, Yu Zhang, Shazlin Shaharudin

**Affiliations:** a Exercise & Sports Science Programme, School of Health Sciences, Universiti Sains Malaysia, Kelantan, Malaysia; b Physical Education and Sport Science (PESS) Department, National Institute of Education, Nanyang Technological University, Singapore; c School of Dental Sciences, Universiti Sains Malaysia, Kelantan, Malaysia

**Keywords:** ACL, biomechanics, human health, sports medicine, rehabilitation

## Abstract

**Objective:**

To evaluate the efficacy of neuromuscular training (NMT) in preventing knee injuries in female team sport athletes and identify dose–response relationships with intervention complexity, compliance, and session parameters.

**Methods:**

This review followed the PRISMA 2020 guidelines. Randomized controlled trials on NMT for injury prevention in female team-sport athletes were retrieved from PubMed, Scopus, Web of Science, EMBASE, and the Cochrane Library up to December 2024. Pooled risk ratios (RRs) with 95% confidence intervals (CIs) were calculated using fixed- or random-effects models depending on heterogeneity. Subgroup analyses examined compliance, session duration, training frequency, and NMT components.

**Results:**

NMT significantly reduced overall knee injury risk by 22% (RR = 0.78, 95% CI: 0.65–0.94, *p* = 0.008) and ACL injury risk by 50% (RR = 0.50, 95% CI: 0.31–0.81, *p* = 0.005). Specifically, high compliance (≥75%) was identified as the most effective factor (RR = 0.67, 95% CI: 0.51–0.87, *p* = 0.004), outperforming program complexity. Sessions lasting longer than 15 min (RR = 0.74, 95% CI: 0.60–0.93, *p* = 0.008) and delivered 2–3 times per week (RR = 0.87, 95% CI: 0.76–0.99, *p* = 0.04) showed significant benefits. Moreover, agility (RR = 0.71, 95% CI: 0.56–0.88, *p* = 0.002) and running mechanics training (RR = 0.80, 95% CI: 0.70–0.93, *p* = 0.003) were independently associated with injury prevention.

**Conclusion:**

NMT reduced knee injury risk in female team sport athletes, with compliance ≥75% indispensable. ≥15-minute sessions delivered 2–3 times weekly can optimise the outcomes. Meanwhile, clinical implementation should prioritize coach-led adherence strategies and evidence-based programs such as FIFA 11+ injury prevention programme (FIFA 11+). Finally, standardising exposure metrics and incorporating sensor-based compliance tracking will enhance scalability.

## 1. Introduction

Knee injuries are among the most common and burdensome musculoskeletal injuries in team sports, particularly in soccer, basketball, and handball [1]. Epidemiological evidence indicated that female athletes experience non-contact knee injuries at rates approximately two to eight times higher than male athletes [2]. These injuries included Anterior Cruciate Ligament (ACL) tears, patellofemoral pain syndrome, meniscal damage, and collateral ligament sprains, with ACL injuries accounting for 40%–60% of these cases [3]. In contrast, reliable pooled estimates for other knee injury subtypes are lacking due to heterogeneous definitions and inconsistent reporting across epidemiological studies. For example, ACL injury incidence among female soccer players ranged from 0.16 to 0.24 per 1,000 athlete-exposures, compared to 0.03 to 0.06 in their male counterparts [1]. Comparable patterns were reported in female basketball and handball athletes [4]. In addition to ACL injuries, knee pathologies such as patellofemoral pain, meniscal lesions, and collateral ligament damage frequently resulted in a prolonged absence from sport, increased risk of reinjury, and a substantially elevated likelihood of early-onset osteoarthritis within 10–15 years following the initial trauma [5].

Biomechanical limitations, such as aberrant landing mechanics (including excessive dynamic knee valgus, limited hip and knee flexion, or asymmetric ground reaction forces during jump-landing), are modifiable risk factors that NMT addresses through enhanced movement control [6,7]. Such deficits can be resolved by improving movement control and enhancing lower limb stability, thereby reducing injury incidence. Specifically, plyometric, strength-based, and eccentric hamstring exercises have been shown to reduce ACL loading by decreasing the duration of the stretch–shortening cycle and limiting anterior tibial shear forces during dynamic actions [8–10]. Concurrently, balance training enhanced proprioceptive acuity, improving joint stability at ground contact [11]. Standard NMT protocols typically include balance exercises, resistance training, agility drills, plyometric movements, and running technique refinement [12,13]. Although NMT has been widely studied, most evidence on its ‘dose’ derives from mixed-sex cohorts rather than female-specific samples [14–16]. One of the few female-focused analyses, Sugimoto et al., showed that weekly training volumes above 30 min reduced ACL injury risk, but this conclusion was based on limited studies and threshold-level comparisons without systematic evaluation of frequency, duration, or cycle length. A recent systematic review further indicated that female athletes may benefit more from NMT, particularly for ACL injury prevention, yet the optimal dose remains uncertain [17]. Thus, further investigation is required to clarify the dose characteristics of NMT in female athletes, and to establish their validity and generalizability [12].

Meta-analytical evidence suggests that NMT can reduce the risk of ACL injury in female athletes by 40% to 70%, based on pooled RR estimates [18,19]. Nevertheless, several methodological limitations remain in the current body of literature [20]. First, most studies focus solely on ACL outcomes and omit other clinically relevant knee injuries. Moreover, intervention heterogeneity is rarely addressed. Considerable variation existed in program content and dosage across trials, yet few studies have systematically examined dose–response relationships, thereby limiting opportunities to optimise preventive strategies.

To address these gaps, this review targeted explicitly female team sport athletes, a population at high risk but underrepresented in current evidence [7].

Previous reviews have predominantly examined ACL injuries in mixed athletic populations, often pooling heterogeneous interventions and limiting clinical applicability [17]. Accordingly, this systematic review and meta-analysis aimed to evaluate the effectiveness of NMT in preventing knee injuries, with a specific focus on ACL injuries, in female team sport athletes. While other acute knee injuries such as meniscal or collateral ligament injuries may also occur, these outcomes were rarely reported in eligible trials. Therefore, the analysis primarily focused on overall knee injury incidence and ACL-specific outcomes. We introduced a novel classification of NMT protocols based on structural complexity, and subgroup analyses were conducted to examine program characteristics such as training frequency, session duration, and compliance. These findings aim to support the development of tailored, evidence-based injury prevention strategies.

## 2. Methodology

### 2.1. Data source

This meta-analysis was prospectively registered with PROSPERO (CRD420251080394) and conducted following the PRISMA 2020 guidelines [21]. A comprehensive literature search was conducted in PubMed, Web of Science, Cochrane Library, Scopus, and EMBASE from inception to 31 December 2024, using both Medical Subject Headings (MeSH) and free-text terms with Boolean operators (AND, OR, NOT). The detailed search strategy is available in Supplementary Appendix A. Duplicate records were removed using EndNote software (Version 21; Clarivate, Philadelphia, PA, USA).

Two independent reviewers (ZR and ZY) screened titles, abstracts, and full texts based on predefined eligibility criteria. Disagreements were resolved through discussion or consulting a third reviewer (SS). Reference lists of included studies and relevant reviews were manually checked for additional eligible records.

### 2.2. Inclusion and exclusion criteria

The inclusion and exclusion criteria are summarised in Table 1. Studies were required to provide sufficient information to derive knee-injury incidence. NMT components were defined as strength, plyometrics, balance/proprioception, agility/change-of-direction, and running/landing mechanics. For subgroup analyses, program complexity was classified a priori as Limited-component NMT (LC-NMT, one or two components) or multicomponent NMT (MC-NMT, ≥3 components), consistent with prior literature. High compliance was defined as ≥75% of prescribed sessions, in line with previous NMT trials [12,22,23].

**Table 1.** Inclusion and exclusion criteria.

Category	Inclusion criteria	Exclusion criteria
Population	Female athletes in organized team sports (aged 12–25 years; adolescence to early adulthood, when knee-injury risk is highest), specifically soccer (football), basketball, volleyball, and handball.	Male-only cohorts or mixed cohorts without sex-disaggregated data; age outside 12–25 years; individual sports or studies without a clear team-sport context
Intervention	Intervention: Neuromuscular training (NMT) program with ≥1 component (see Methods for operational definition)	Programmes without neuromuscular elements, such as stretching-only or aerobic training
Comparator	Non‑NMT control or usual warm‑up/practice	Studies without a comparator
Outcome	Sport-related knee injuries were included, primarily ACL ruptures; other acute injuries, such as meniscal or collateral ligament damage, were infrequently reported and thus not analysed separately.	Non‑knee injuries or studies reporting only chronic/overuse conditions
Language	English‑language, accessible full text	Non‑English publications or inaccessible full text
Study design	Randomized controlled trials (peer‑reviewed full text)	Non‑randomized studies, observational designs, case series, reviews, conference abstracts

### 2.3. Data extraction

Two independent reviewers (ZR and ZY) extracted data using a predefined Microsoft Excel template. Extracted variables included study characteristics (author, year, journal, sport type), participant characteristics (age, competitive level, menstrual cycle status if reported), intervention details (NMT classification, components, frequency, duration), and outcomes (injury counts, athlete exposure, injury subtypes). Compliance data were extracted when reported; studies were classified as high (≥75%) or low (<75%) compliance based on training session completion rates. Discrepancies were resolved by consensus with a third reviewer (SS), following Cochrane guidelines. For subgroup analyses, interventions were categorised as LC-NMT (1–2 components), typically representing specialised or theme-based training protocols, MC-NMT (≥3 components), or structured neuromuscular programs (SSNPs). Although MC-NMT and SSNPs share several multicomponent features, they were treated as distinct groups due to program standardisation and design differences. This classification facilitated interpretation and enhanced statistical power.

### 2.4. Data analysis and processing

Meta-analyses were conducted using R (v4.4.3; R Foundation for Statistical Computing) within RStudio (v2024.12.1-563; Posit). Studies with incomplete outcome data were excluded from the analysis. Such data referred to missing key information, for example, the number of knee or ACL injuries or the corresponding athlete-exposure data required for risk-ratio calculation. Sensitivity analyses were subsequently performed to assess the robustness of the results. Injury outcomes were binary, and pooled effect sizes were reported as RRs with 95% CIs. Heterogeneity was assessed using the I^2^ statistic and Cochran’s Q test, and classified as low (*I*^2^ < 25%), moderate (25%–50%), or high (>50%) [24]. A random-effects model was applied when heterogeneity was substantial (I^2^ ≥ 50% or Q-test *p* < 0.10); otherwise, a fixed-effects model was used. A leave-one-out sensitivity analysis evaluated the influence of individual studies on pooled estimates. Publication bias was assessed by visual inspection of the funnel plot and statistically evaluated using Egger’s regression test. Meta-regression examined the association between compliance levels and effect size. Subgroup analyses investigated the influence of training characteristics on injury prevention efficacy, including frequency, session duration, intervention length, and compliance. Studies were stratified according to reported values for each variable to facilitate comparative evaluation of RR estimates across categories. This approach provided practical insights into how program design variations affect intervention outcomes, without explicitly modeling continuous dose–response relationships.

## 3. Results

### 3.1. Literature search results

A systematic literature search was conducted across five electronic databases: PubMed, Web of Science, Scopus, Embase, and Cochrane Library. This search retrieved 2,326 records in total. After removing 1702 duplicates using EndNote 21 software, 624 unique articles remained for title and abstract screening.

Two reviewers (ZR and ZY) independently screened titles and abstracts based on predefined eligibility criteria. Any discrepancies were resolved through discussion with a third reviewer (SS) until a consensus was reached.

Overall, 558 records were excluded. Specifically, 272 were excluded because of non-randomized or quasi-experimental designs; 138 were non-human studies, narrative reviews, or conference abstracts; and 148 did not meet the predefined intervention criteria.

A total of 66 full-text articles were retrieved for further evaluation. However, 28 articles could not be accessed in full text despite comprehensive database and interlibrary searches, and attempts to contact the authors. Among the remaining 38 studies, 27 were excluded: 17 had incomplete outcome data, for example missing injury counts or insufficient athlete exposure information required to calculate risk ratios, and 10 had data that could not be converted for quantitative analysis.

**Figure 1.**
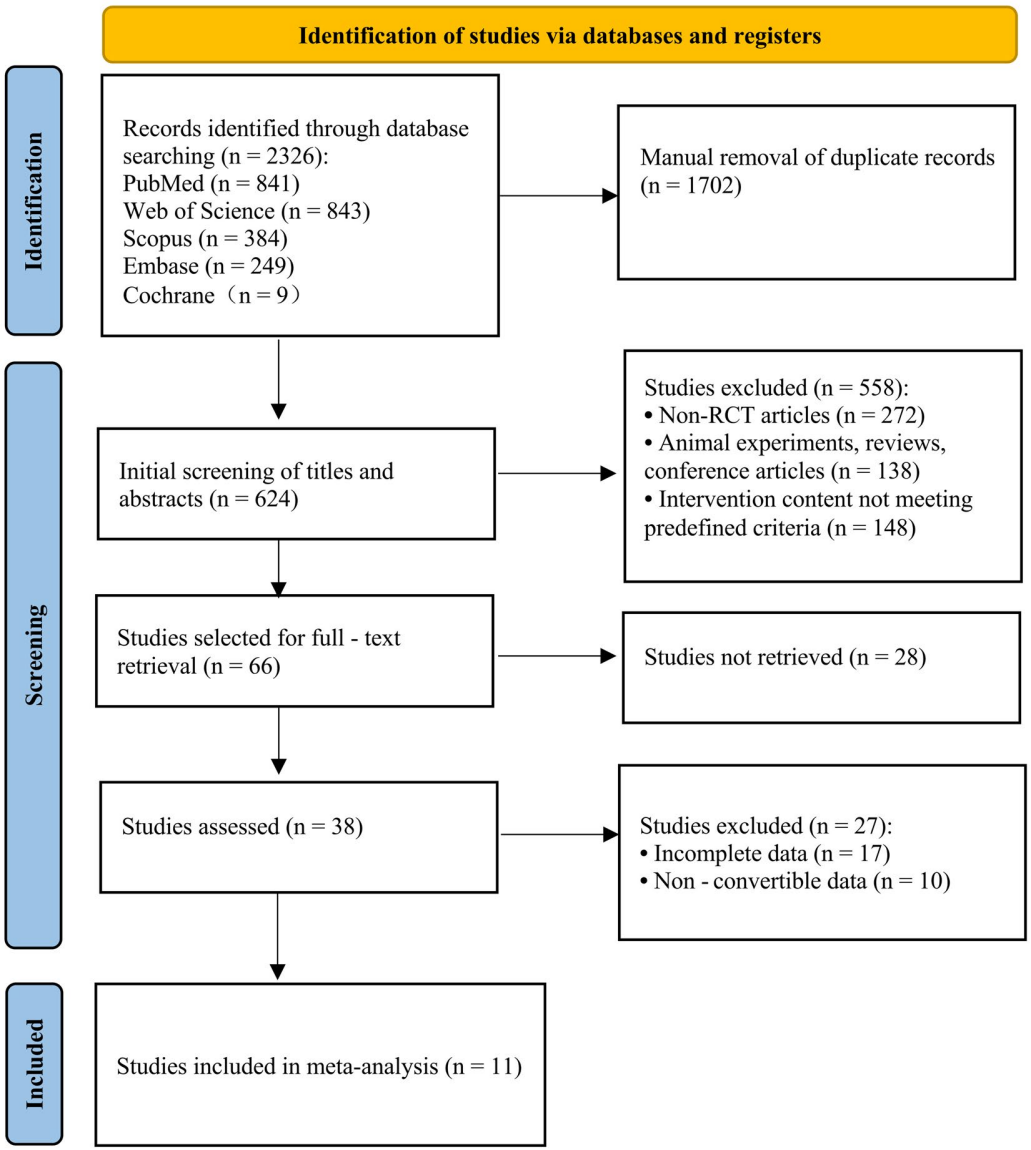
PRISMA flow diagram of the search strategy and study selection.

Ultimately, 11 RCTs fulfilled the inclusion criteria and were included in the meta-analysis, as shown in Figure 1.

### 3.2. Characteristics of included studies

Table 2 summarises the characteristics of the 11 studies investigating NMT or injury prevention programs targeting knee injury in youth and adolescent athletes [23,25–34].

**Table 2.** Data extraction from included articles.

Author	Total sample size (EG/CG)	Age (years)	Population (sport/athlete level)	Session duration (min)	Exercise frequency (times/week)	Intervention duration (weeks)	Compliance classification	Outcome indicator
Bonato et al. (2018)	160 (86/74)	EG (20 ± 2); CG (20 ± 1)	Italian football/basketball/volleyball players; college-level	30	4	32	High compliance	Knee joint injury
Foss et al. (2018)	474 (259/215)	14 ± 1.7	US football/basketball/volleyball players; middle school and high school	10–25	2–3	one season	High compliance	Knee joint injury; ACL
Gilchrist et al. (2008)	1435 (583/852)	19.88	US NCAA Division I women’s football players; collegiate	20–30	3	12	Low compliance	Knee joint injury; ACL
Heidt et al. (2000)	300 (42/258)	14–18	US football players; high school	Unclear	3	7	Unclear	Knee joint injury; ACL
Labella et al. (2011)	1492 (737/755)	EG (16.19 ± 1.53); CG (16.22 ± 1.06)	Chicago public high school football and basketball players; high school	20	unclear	one season	High compliance	Knee joint injury; ACL
Soderman et al. (2000)	140 (62/78)	EG (20.4 ± 4.6); CG (20.5 ± 5.4)	Swedish football players; sub-elite club	10–15	3	one season	High compliance	Knee joint injury; ACL
Soligard et al. (2008)	1892 (1055/837)	13–17	Norwegian football players; Youth club	20	2–5	32	High compliance	Knee joint injury
Steffen et al. (2008)	2020 (1073/947)	13–17	Norwegian U17 football league players; Youth club	20	2–3	16	Low compliance	Knee joint injury; ACL
Steffen et al. (2013)	158 (78/80)	13–18	Norwegian football players; Youth club	20	2–3	16	High compliance	Knee joint injury
Walden et al. (2012)	4564 (2479/ 2085)	EG (14.0 ± 1.2); CG (14.1 ± 1.2)	Swedish football players; Adolescent youth club	15	2	28	Low compliance	Knee joint injury
Zebis et al. (2015)	40 (20/20)	EG 15.9; CG 15.6	Danish football and handball players; Sports College	15	3	12	High compliance	Knee joint injury; ACL

Note: This table summarizes key characteristics of neuromuscular training (NMT) interventions in female team-sport athletes. EG: experimental group; CG: control group; ACL: anterior cruciate ligament. Session duration refers to the length of each training session; exercise frequency indicates how often training was performed per week. Compliance classification is based on the proportion of prescribed training sessions completed, with high compliance defined as ≥75% completion and low compliance as <75% completion. ‘Unclear’ indicates data not reported in the original article.

Sample sizes ranged from 40 to 4564, with eight studies focused on football and three on basketball, volleyball, or handball. The ages ranged from 12 to 25 years.

Interventions included 10–30 min sessions delivered 2–5 times weekly, from 7 weeks to one season. Approximately six studies applied NMT 3 times weekly as part of standard warm-ups.

All studies reported knee injury outcomes; eight articles explicitly included ACL injury data, while only 2 small-scale trials focused on biomechanical mechanisms. Athlete cohorts ranged from middle- and high-school students to collegiate NCAA Division I players and youth or sub-elite club athletes, thus covering a spectrum from adolescent to competitive levels.

### 3.3. NMT components included in each intervention and their classification

Table 3 summarises the NMT components included in each intervention and their classification by intervention type. Five core components were evaluated across all 11 studies: agility, balance, plyometrics, running mechanics, and strength. Components included per intervention ranged from 20% to 100%, with seven studies (63.6%) incorporating at least four components. Strength was the most frequently included component (10 of 11 studies, 90.9%), followed by balance and plyometrics (each included in nine studies, 81.8%); agility and running mechanics were included in seven studies (63.6%). Only three studies integrated all five components. Interventions were classified into three types according to structural complexity: MC-NMT, incorporating three or more components; LC-NMT, including one or two related components within the same training domain (such as balance-only programs or narrow combinations like balance + strength); and SSNPs, such as the FIFA 11+ injury prevention program (FIFA 11+).

**Table 3.** Summary of NMT components across interventions.

Study ID	Author (year)	Intervention type	Agility	Balance	Plyometrics	Running mechanics	Strength	Proportion of components included (%)
1	Bonato et al. (2018)	Bodyweight NMT	Yes	Yes	Yes	No	Yes	80% (4 of 5 components)
2	Foss et al. (2018)	Core-Focused NMT	Yes	Yes	Yes	Yes	Yes	100% (5 of 5 components)
3	Gilchrist et al. (2008)	Preventive Plyometric-Neuromuscular Program	Yes	No	Yes	Yes	Yes	80% (4 of 5 components)
4	Heidt et al. (2000)	Preseason Conditioning Program	Yes	No	Yes	Yes	Yes	80% (4 of 5 components)
5	LaBella et al. (2011)	Balance Board Training	No	Yes	No	No	Yes	40% (2 of 5 components)
6	Soderman et al. (2000)	Neuromuscular Warm-up	No	Yes	No	No	No	20% (1 of 5 components)
7	Soligard et al. (2008)	FIFA 11+	Yes	Yes	Yes	Yes	Yes	100% (5 of 5 components)
8	Steffen et al. (2008)	FIFA 11 (The 11)	No	Yes	Yes	Yes	Yes	100% (5 of 5 components)
9	Steffen et al. (2013)	FIFA 11+	Yes	Yes	Yes	Yes	Yes	100% (5 of 5 components)
10	Walden et al. (2012)	Multimodal NMT	No	Yes	Yes	Yes	Yes	80% (4 of 5 components)
11	Zebis et al. (2015)	Progressive Neuromuscular Strength Training	Yes	Yes	Yes	No	Yes	80% (4 of 5 components)
Total (%)			63.6%	81.8%	81.8%	63.6%	90.9%	

Note: ‘Yes’ indicates that the component was included in the intervention; ‘No’ indicates that it was not. Interventions were classified into three categories based on structural characteristics: (A) Multicomponent NMT (MC-NMT), comprising three or more components (agility, balance, plyometrics, strength, running mechanics, or core stability); (B) Limited-component NMT (LC-NMT), including one or two related components within the same training domains; and (C) Standardised Structured Neuromuscular Programs (SSNPs), referring to protocol-based interventions with fixed progression, such as FIFA 11+ or FIFA 11. ‘Components Used (%)’ refers to the proportion of the five predefined components present in each intervention. ‘Total (%)’ denotes the percentage of studies (*n* = 11) in which each specific component was included.

### 3.4. Methodological quality

The methodological quality of the 11 included studies was evaluated using the Cochrane Risk of Bias tool (RoB 2.0).

**Figure 2.**
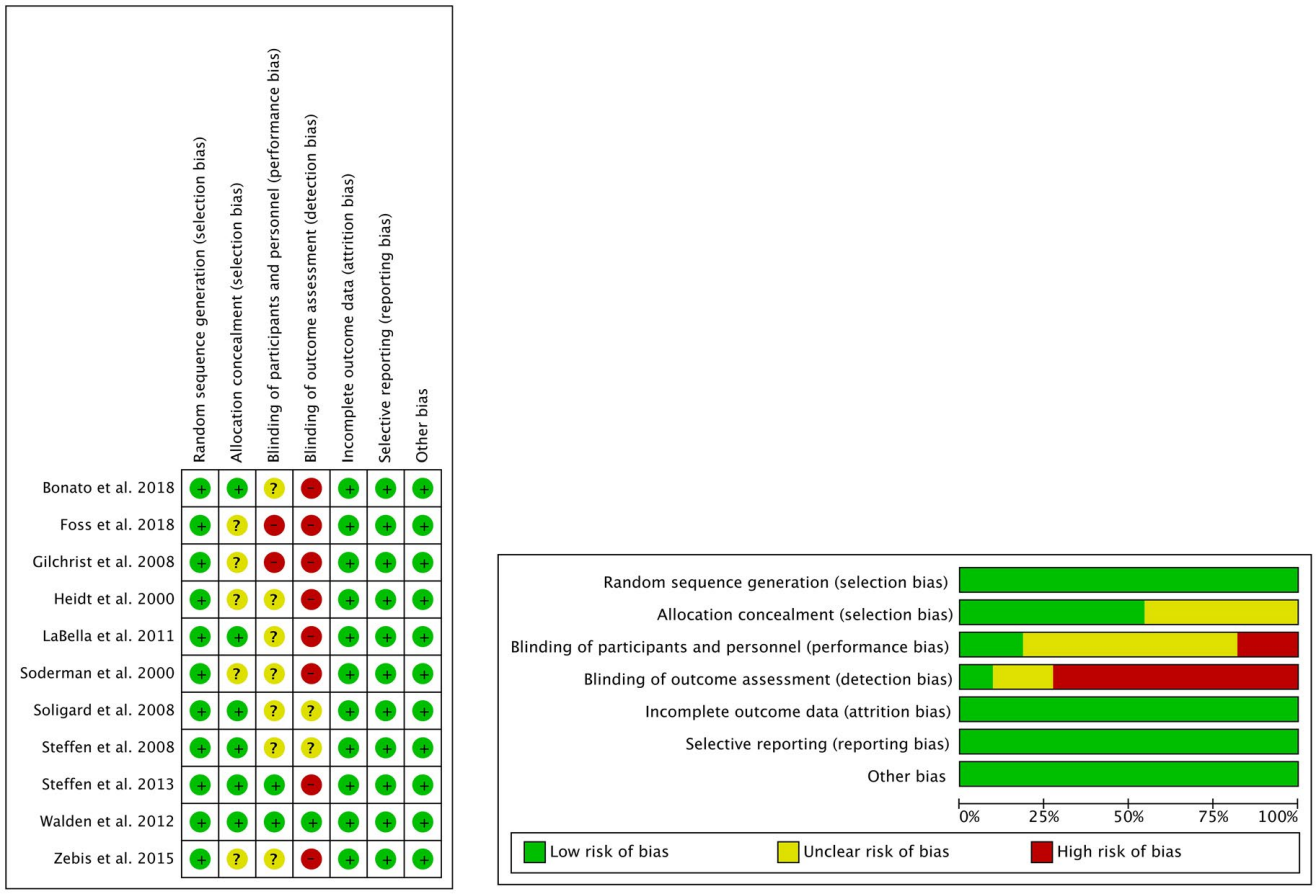
Overall risk of bias overview.

- Random sequence generation (selection bias): All 11 studies were rated low risk.
- Allocation concealment (selection bias): six studies were considered low risk, while five were deemed to have an unclear risk.
- Blinding of participants and personnel (performance bias): two studies were considered low risk, two were high risk, and seven were classified as unclear risk.
- Blinding of outcome assessment (detection bias): eight studies were judged to be at high risk, two at unclear risk, and only one at low risk.
- Incomplete outcome data (attrition bias): All studies were assessed as low risk.
- Selective reporting (reporting bias): All studies were classified as low risk.
- Other sources of bias: No additional sources were identified; all studies were considered low-risk in this domain, as shown in Figure 2.

### 3.5. Evaluation of meta-analysis results

#### 3.5.1. Overall effect test

This meta-analysis was conducted on 11 studies, including 6,474 participants in the intervention group and 6,201 in the control group. Moderate heterogeneity (*I*^2^ = 41.4%, *p* = 0.07) justified the use of a random-effects model. The pooled RR for overall knee injuries was 0.78 (95% CI: 0.65–0.94, *p* = 0.008), demonstrating a statistically significant reduction in knee injury risk associated with NMT.

**Figure 3.**
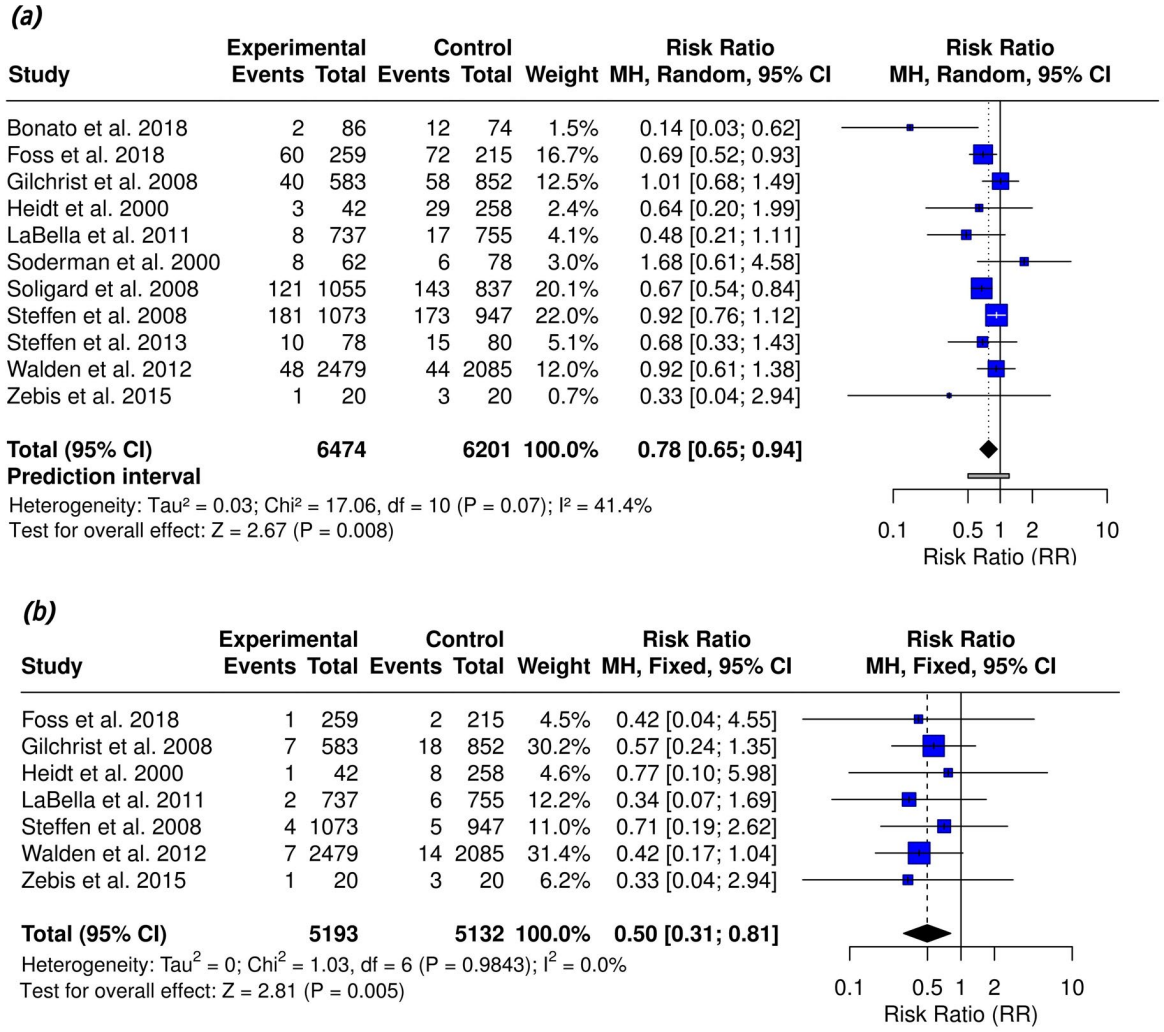
Forest plots of NMT interventions on knee and ACL injury risk.

A further analysis focusing on ACL injuries, comprising seven studies with 10,325 participants, produced a pooled RR of 0.50 (95% CI: 0.31–0.81; *I*^2^ = 0%, *p* = 0.005), indicating a consistent protective effect of NMT in preventing ACL injuries with minimal heterogeneity as presented in Figure 3.

#### 3.5.2. Subgroup analysis results

**Figure 4.**
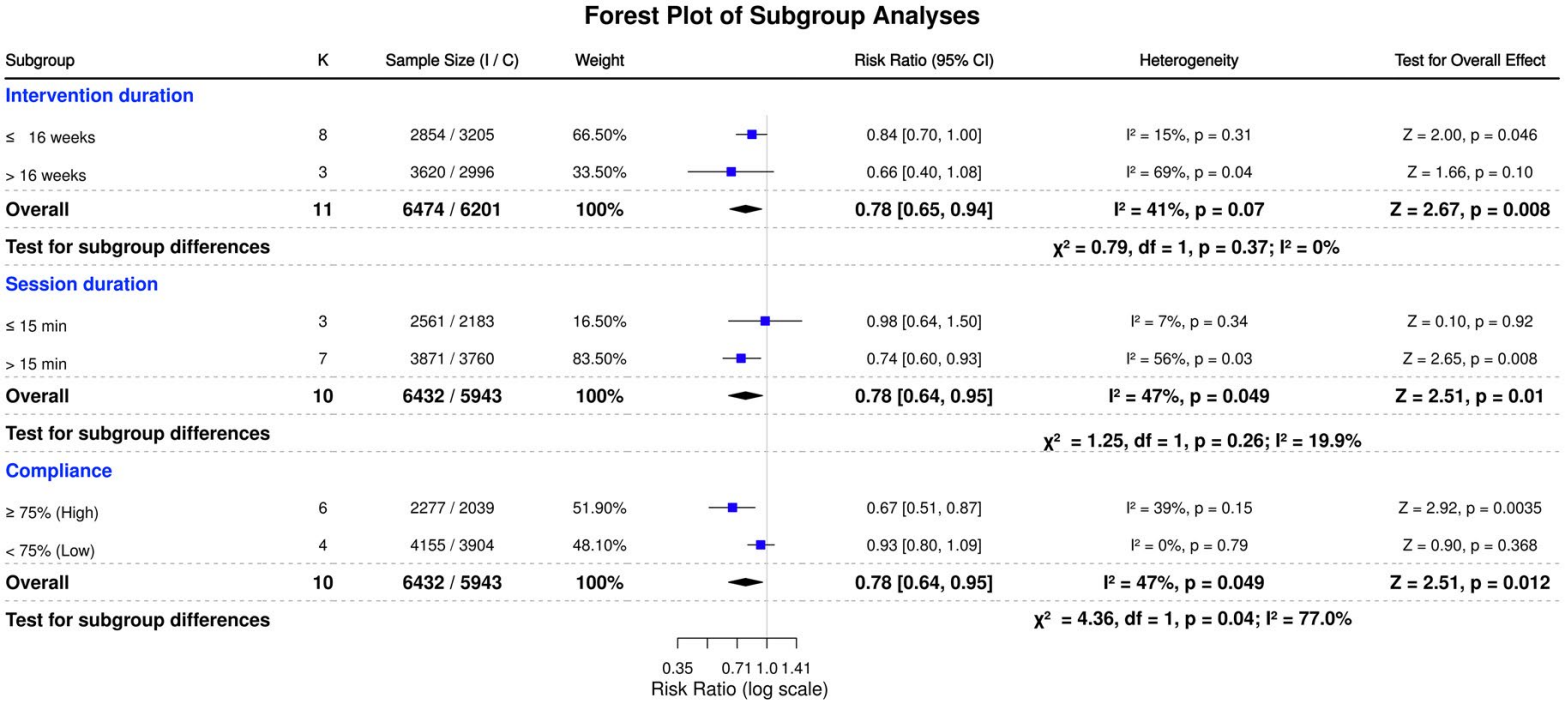
Forest plots of NMT interventions on knee injury risk: subgroup analyses by intervention duration, session duration, and compliance.

**Figure 5.**
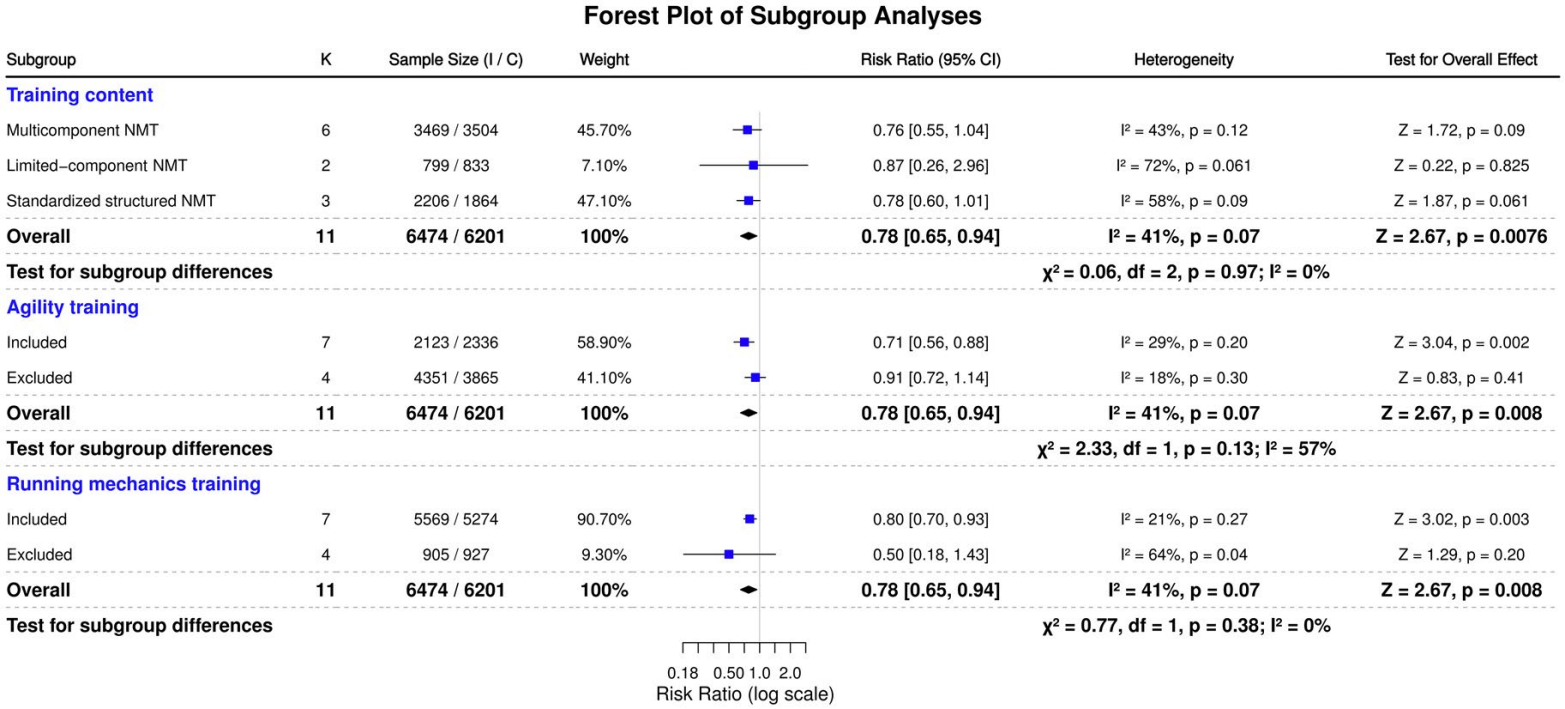
Forest plot of the effect of NMT interventions on knee injury risk: Subgroup analysis by training type and component.

To identify sources of heterogeneity, subgroup analyses assessed seven moderators: intervention duration, session duration, compliance level, intervention type, training frequency, and two key components (agility and running mechanics). These analyses quantified how program characteristics influenced NMT effectiveness in reducing knee injury risk, as illustrated in Figures 4 and 5.

##### 3.5.2.1. Duration of intervention

Subgroup analysis by intervention duration (11 studies) showed a statistically significant 16% reduction in injury risk for programs lasting ≤ 16 weeks (RR = 0.84, 95% CI 0.70–1.00, *p* = 0.046; *I*^2^ = 15%). In contrast, interventions exceeding 16 weeks yielded a non-significant 34% risk reduction (RR = 0.66, 95% CI 0.40–1.08, *p* = 0.10), with substantial heterogeneity (*I*^2^ = 69%). The between-subgroup difference was not significant (*p* = 0.37), suggesting no clear effect modification by intervention duration.

##### 3.5.2.2. Session duration

Subgroup analysis by session duration (10 studies) showed that injury risk was significantly reduced when NMT sessions lasted longer than 15 min (RR = 0.74, 95% CI: 0.60–0.93, *p* = 0.008; *I*^2^ = 56%). In contrast, shorter sessions (≤15 min) were not associated with a significant reduction in injury risk (RR = 0.98, 95% CI: 0.64–1.50, *p* = 0.92; *I*^2^ = 7%). The difference between subgroups was insignificant (*p* = 0.26), suggesting no clear interaction between session duration and intervention effectiveness.

##### 3.5.2.3. Compliance level

Subgroup analysis based on intervention compliance (10 studies) revealed a significant 33% reduction in injury risk among studies with high compliance (≥75%) (RR 0.67, 95% CI 0.51–0.87, *p* = 0.004; *I*^2^ = 39%). In contrast, studies with low compliance (<75%) showed no significant risk reduction (RR 0.93, 95% CI 0.80–1.09, *p* = 0.37), with minimal heterogeneity (*I*^2^ = 0%). The difference between subgroups was statistically significant (*p* = 0.04; *I*^2^ = 77%), indicating that compliance served as a critical moderator of intervention effectiveness.

##### 3.5.2.4. Intervention type

Subgroup analyses stratified by intervention type (11 studies) revealed that MC-NMT programs showed a trend toward risk reduction (RR = 0.76; 95% CI: 0.55–1.04; *I*^2^ = 43%, *p* = 0.09), although this did not reach statistical significance. Structured programs similarly demonstrated a potential risk reduction (RR = 0.78; 95% CI: 0.60–1.01; *I*^2^ = 58%, *p* = 0.061). Conversely, LC-NMT did not yield a statistically significant reduction in injury risk (RR = 0.87; 95% CI: 0.26–2.96; I^2^ = 72%, *p* = 0.825). No significant differences were observed among subgroups (*p* = 0.97).

##### 3.5.2.5. Incorporation of agility training

Subgroup analyses based on the inclusion of agility training (11 studies) showed that interventions incorporating agility exercises were associated with a statistically significant reduction in knee injury risk (RR = 0.71; 95% CI: 0.56–0.88; *I*^2^ = 29%, *p* = 0.002). In contrast, interventions that excluded agility training did not demonstrate a significant effect (RR = 0.91; 95% CI: 0.72–1.14; *I*^2^ = 18%, *p* = 0.41). However, subgroup differences were not statistically significant (*p* = 0.13).

##### 3.5.2.6. Running mechanics training

When stratified by the inclusion of running mechanics training (11 studies), programs incorporating this component significantly reduced injury risk (RR = 0.80; 95% CI: 0.70–0.93; I^2^ = 21%, *p* = 0.003). On the other hand, interventions that excluded running mechanics training did not reach statistical significance (RR = 0.50; 95% CI: 0.18–1.43; *p* = 0.20). The between-group difference was not statistically significant (*p* = 0.38).

##### 3.5.2.7. Training frequency

For programs with a training frequency of 2–3 sessions per week, the pooled analysis of 8 studies demonstrated a significant 13% reduction in injury risk (RR = 0.87; 95% CI: 0.76–0.99; I^2^ = 0%, *p* = 0.04), indicating negligible between‐study heterogeneity.

### 3.6. Sensitivity analysis of pooled effect

A leave-one-out sensitivity analysis was performed to assess the robustness of the pooled effect estimates. The pooled RRs ranged from 0.75 to 0.81, with all 95% confidence intervals excluding the null, thereby supporting the stability of the observed protective association. Notably, heterogeneity was markedly reduced (*I*^2^ = 23%) upon excluding Bonato et al., which exhibited outlier characteristics. The overall association remained stable after excluding studies with limited sample sizes [35] and imprecise estimates [21]. No individual study disproportionately influenced the summary effect, indicating methodological robustness. Consistent findings were obtained under both fixed-effect and random-effects models, further validating the reliability of the results.

### 3.7. Assessment of publication bias

**Figure 6.**
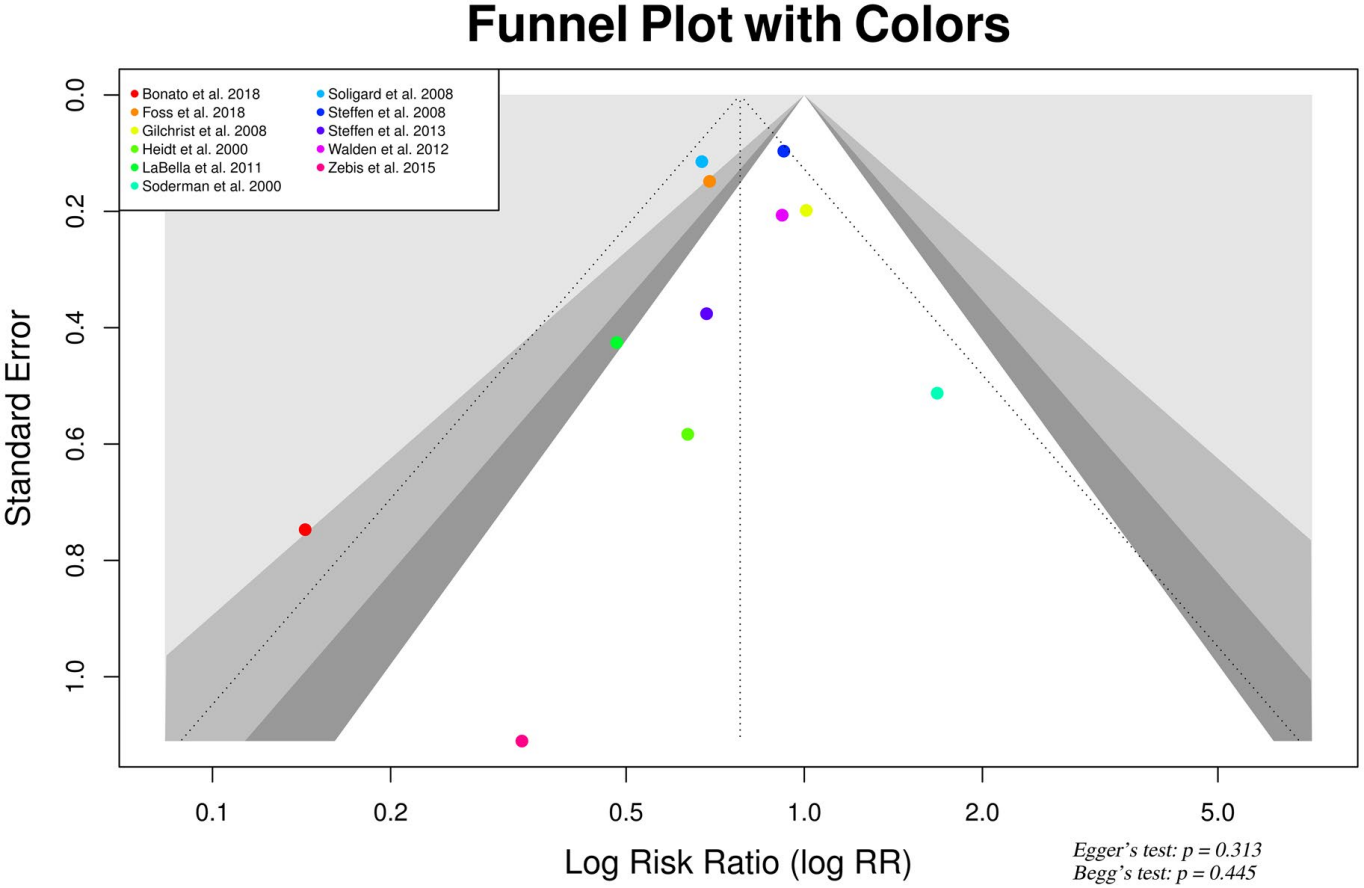
Funnel plot analysis.

Publication bias was evaluated through both visual inspection and statistical methods. The funnel plot, including 11 studies, exhibited slight asymmetry, characterized by a deficiency of small-sample studies on the right side (smaller effect sizes), suggesting possible small-study effects or selective non-publication of null results. Egger’s regression test detected no significant asymmetry (*t* = −1.07, *p* = 0.313), and Begg’s rank correlation test similarly found no evidence of publication bias (Kendall’s tau = −0.20, *p* = 0.445), indicating a low probability of substantial bias as shown in Figure 6.

### 3.8. Effect of compliance on knee injury risk

Meta-regression was performed to assess the effect of compliance level on intervention effectiveness across 10 studies. The model showed a significant moderator effect of compliance (QM(1) = 7.68, *p* = 0.0056).

The intercept, representing the low compliance group, was not statistically significant (estimate = −0.0736, SE = 0.0814, *p* = 0.366). The coefficient for the high compliance group was significant and negative (estimate = −0.3237, SE = 0.1168, *p* = 0.006).

Residual heterogeneity was negligible (*τ*^2^ < 0.001, *I*^2^ ≈ 0%), suggesting that most between-study variation was explained by compliance level, though this should be interpreted cautiously given the small sample size (*k* = 10).

**Figure 7.**
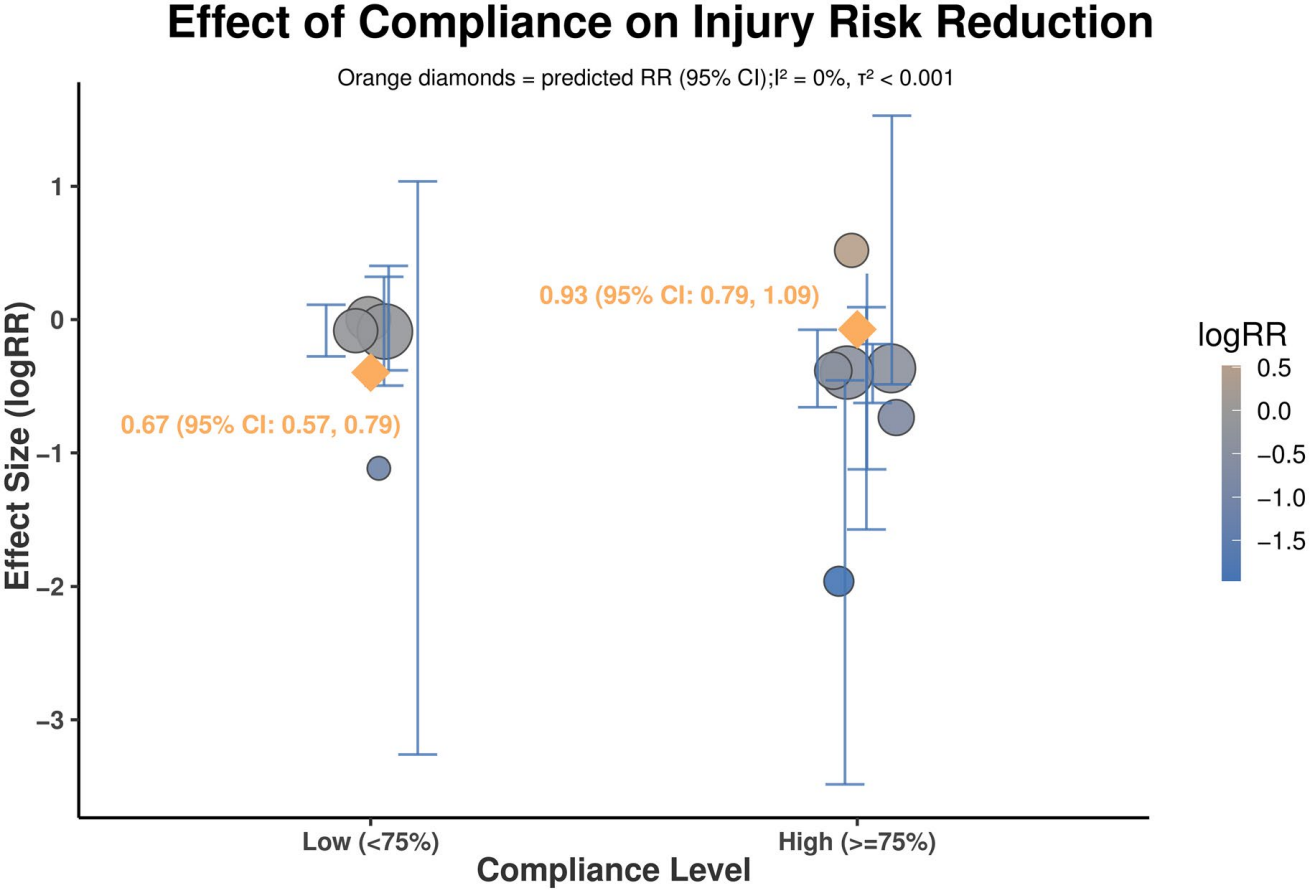
Meta-regression analysis of the association between compliance level and knee injury risk.

Predicted logRR were −0.074 (95% CI: −0.233, 0.086) for low compliance and −0.398 (95% CI: −0.553, −0.095) for high compliance as presented in Figure 7.

## 4. Discussion

### 4.1. Major findings

This systematic review provided level I evidence that NMT programs significantly reduced overall knee injury risk in female team-sport athletes by 22% (RR = 0.78, 95% CI: 0.65–0.94, *p* = 0.008). A focused analysis of ACL injuries (*n* = 10,325) revealed an even greater protective effect, with a 50% reduction in risk (RR = 0.50, 95% CI: 0.31–0.81, *p* = 0.005), underscoring the anatomical specificity and critical preventive potential of NMT for ACL injury. Subgroup analyses indicated significant effects of compliance (≥75%), session duration (>15 min), training frequency (2–3 sessions per week), and inclusion of agility and running-mechanics components. In contrast, multicomponent and structured protocols showed only non-significant trends (*p* > 0.05), and LC-NMT was ineffective.

### 4.2. Subgroup analyses and modifying factors

#### 4.2.1. Intervention compliance as the primary moderator

Compliance emerged as the most influential determinant of NMT efficacy. Participants with high compliance (≥75%) exhibited a 33% reduction in injury risk (RR = 0.67, 95% CI: 0.51–0.87, *p* = 0.004), whereas lower compliance conferred no protective benefit (RR = 0.93, 95% CI: 0.80–1.09, *p* = 0.37). This finding aligns with Steffen et al. (2013), who documented a 58% lower injury incidence among highly adherent athletes (IRR = 0.43), establishing a dose-dependent relationship between compliance and prevention efficacy [36].

Notably, this compliance-dependent effect size difference exceeded effect sizes for all structural factors. Multicomponent NMT yielded a 24% risk reduction (RR = 0.76, 95% CI: 0.55–1.04), which was not statistically significant. Similarly, standardised protocols such as FIFA 11+ demonstrated comparable non-significant effects (RR = 0.78, 95% CI: 0.60–1.01, *p* > 0.05) [37]. These results indicated that NMT success depends more on implementation fidelity than program design. Even theoretically optimal interventions may fail without adequate compliance [20].

##### 4.2.2. Intervention type

Multicomponent interventions (≥3 neuromuscular elements) showed a clinically relevant 24% reduction in injury risk (RR = 0.76), although this result was not statistically significant (*p* = 0.09). Standardised programs such as FIFA 11+ showed similar trends (RR = 0.78), whereas Limited-component NMT had no effect (RR = 0.87, 95% CI: 0.26–2.96, *p* = 0.82). This trend is consistent with prior reviews favouring multifactorial programs, although our analysis could not confirm a significant benefit [22].

##### 4.2.3. Intervention duration and session length

Session duration demonstrated a dose–response relationship. Protocols exceeding 15 min per session were associated with reduced injury risk (RR = 0.74, 95% CI: 0.60–0.93, *p* = 0.008), whereas sessions ≤15 min were ineffective (RR = 0.98, 95% CI: 0.64–1.50, *p* = 0.92). This pattern is consistent with prior work [14]. For intervention duration, programs ≤16 weeks achieved statistical significance (RR = 0.84, 95% CI: 0.70–1.00, *p* = 0.046) with low heterogeneity. In contrast, longer programs (greater than 16 weeks) showed a non-significant trend toward greater risk reduction (RR = 0.66, 95% CI: 0.40–1.08, *p* = 0.10) along with considerable heterogeneity (I^2^ = 69%). The reduced compliance during these extended interventions likely weakened their effectiveness [35].

##### 4.2.4. Component-specific effects

Agility training correlated with a significant 29% reduction in knee injury risk (RR = 0.71, 95% CI: 0.56–0.88). Biomechanical evidence indicated that agility-focused NMT improved lower-limb control during high-risk manoeuvres such as cutting and pivoting, major contributors to ACL injuries [38]. Programs excluding agility training produced non-significant effects (RR = 0.91, *p* = 0.38) [39].

Running mechanics training significantly reduced injury risk (RR = 0.80, 95% CI: 0.70–0.93). Moreover, programs without this component showed a larger point estimate (RR = 0.50) that was non-significant (*p* = 0.20) with wide confidence intervals, reflecting substantial heterogeneity. These findings underscored the need to address proprioceptive deficits and movement pattern deviations [40].

##### 4.2.5. Training frequency

Inconsistent reporting precluded formal subgroup analysis beyond 2–3 sessions weekly. However, descriptive analysis revealed that programs administered 2–3 times weekly significantly reduced injury risk by 13% (RR = 0.87, 95% CI: 0.76–0.99, *p* = 0.04; I^2^ = 0%) [14]. This analysis supported the consensus that moderate frequency sufficed to induce neuromuscular adaptations when fidelity was maintained. Future research should determine whether higher frequencies enhance prevention or compromise compliance.

### 4.3. Associations between training-dose metrics and efficacy

Subgroup analyses identified significant associations between training-dose metrics (compliance, session duration, and training frequency) and knee injury prevention, consistent with trends in Steib et al., who emphasized the interplay of intensity, frequency, and compliance in determining NMT outcomes. High compliance (≥75%) was linked to a 33% risk reduction, sessions >15 min conferred a 26% risk reduction, and 2–3 sessions/week were associated with a 13% risk reduction.

However, the absence of formal dose-response modelling (such as meta-regression) limited causal inference [41]. Substantial variability in reporting total program exposure (minutes/week) underscored the need for standardised dose metrics and future high-quality RCTs on frequency, intensity, and periodization. Formal meta-regression was precluded due to inconsistent reporting of exact session counts, weekly volume (minutes/week), and intensity metrics across studies. Future trials should adopt uniform dose descriptors to enable pooled quantitative modelling.

### 4.4. Physiological mechanisms

The protective effects of NMT are thought to arise from the multidimensional modulation of neuromuscular control and biomechanical parameters, as demonstrated in prior biomechanical and neurophysiological studies conducted predominantly in young female athletes. Previous kinematic studies have shown [6] that NMT can reduce dynamic knee valgus angles by 12° ± 3° (*p* < 0.001), optimise muscle activation timing (such as vastus medialis obliquus–vastus lateralis synchrony), and decrease anterior tibial shear forces *via* shortened muscle latency (50–100 ms), thereby reducing ACL loading. Moreover, athletes with prior ACL injury exhibited greater motor pattern corrections, reflecting use-dependent neuroplasticity. This aligns with findings by Padua et al., who reported cortical reorganization in female athletes following balance-based laboratory interventions. Such proprioceptive enhancement and dynamic stability improvements, reported in earlier experimental trials [42], may further underpin the efficacy of NMT^6^. Agility training may provide superior preventive benefits by integrating reactive motor control, rapid neuromuscular adjustments, and visuomotor coordination during unanticipated cutting and pivoting. These demands on sensorimotor integration enhance anticipatory postural adjustments and joint stability, which may explain its greater preventive effect compared to isolated balance or plyometric drills [7,38]. Biological maturation may also modulate the preventive effects of NMT in female athletes. Hormonal fluctuations during the menstrual cycle, particularly around menarche and the ovulatory phase, have been linked to increased ligamentous laxity and reduced joint stability [43]. Pubertal growth spurts contribute to neuromuscular control deficits and altered biomechanics [44,45], further elevating ACL injury risk. Longitudinal evidence indicates that neuromuscular control evolves with maturation stages [46,47], highlighting the need to tailor NMT protocols to athletes’ maturational status [48,49].

### 4.5. Limitations

Most included trials reported only overall knee or ACL-specific outcomes, limiting interpretation of other injury subtypes. Although this review incorporated high-quality randomised trials, several limitations should be acknowledged. Most studies focused on football, limiting generalisability to other team sports. All sports were therefore pooled rather than analysed separately, because the small number of trials in basketball, volleyball, and handball precluded adequately powered sport-specific subgroup analyses. Heterogeneous reporting of intervention doses and compliance (self-report vs. coach-supervised) introduced statistical heterogeneity and performance bias, with self-reported compliance likely overestimating engagement [50]. The lack of stratified analyses by prior knee injury history limited insights into secondary prevention, where athletes might have altered neuromechanical baselines [51]. High heterogeneity in LC-NMT (*I*^2^ = 72%) and long-duration programs (*I*^2^ = 69%) highlighted the need for homogeneous study designs. Moreover, most included studies did not account for athletes’ habitual physical activity outside of NMT (such as aerobic exercise, stretching, or conditioning), which may have acted as a confounding factor. Furthermore, 28 potentially eligible articles could not be retrieved in full text despite extensive searches and author contact, while 17 full-text studies were excluded due to incomplete outcome data. Both exclusions may have introduced bias and should be considered when interpreting the findings.

### 4.6. Clinical implications and future directions

Compliance is the cornerstone of successful NMT-based injury prevention. As such, clinicians should prioritise strategies that consistently achieve ≥75% compliance, such as coach-led delivery, team-based accountability, and wearable feedback systems, because high compliance independently predicted a 33% injury risk reduction, which exceeded the effect of program complexity and was comparable to or greater than the effect of session length [51–53]. Program structure, session duration (greater than 15 min), and frequency (2–3 sessions per week) were significant factors, but their effectiveness depended on consistent implementation. Even multicomponent or standardised programs, such as FIFA 11+, may fail to produce a clinical benefit without sufficient compliance. However, their structured design can still enhance implementation fidelity by providing fixed progressions and consistent delivery formats, thereby reducing methodological variability across studies, although their independent effect was not significant in our analysis. Integrating NMT components into regular warm-up routines may further optimise time efficiency and adherence, as these programs can be delivered within the first 15–20 min of standard training sessions. Seasonal timing may also influence program effectiveness: pre-season implementation allows athletes to adapt neuromuscular patterns under lower competitive stress, whereas in-season programs face challenges from fatigue and scheduling; future research should examine this factor explicitly. To facilitate real-world application, future programs should embed behaviorally-informed mechanisms (for example, gamification, scheduling consistency, mobile nudges) before optimising technical components. In youth sports, practical strategies that are low-cost and scalable, like peer monitoring and digital checklists, might be more effective than relying on AI-based personalisation. Finally, standardised compliance reporting—*via* objective tools such as inertial sensors—should be incorporated into trial designs to enable pooled analyses and guide dose–response modelling. Scientific principles must complement biomechanical theory to achieve lasting injury reduction.

## 5. Conclusion

This meta-analysis confirmed that NMT significantly reduces the risk of knee injuries in female team-sport athletes, demonstrating a 22% overall risk reduction (RR = 0.78; 95% CI: 0.65–0.94) and a 50% reduction in ACL injuries (RR = 0.50; 95% CI: 0.31–0.81). Compliance rates of ≥75% were the most influential determinant of intervention efficacy, resulting in a 33% decrease in injury risk (RR = 0.67), independent of program complexity. Agility and running mechanics components, together with session durations exceeding 15 min, were associated with optimal outcomes. A training frequency of 2 to 3 sessions per week was sufficient to maintain effectiveness.

For clinical implementation, the following strategies were recommended: (1) Enhancing adherence through coach-supervised delivery and formal accountability structures; (2) Prioritising evidence-based programs that emphasise sport-specific agility exercises; (3) Initiating interventions during the early season (within 16 weeks) may maximise preventive benefit. Future studies should adopt an analysis of dose parameters (such as total session minutes per week) and employ sensor-based adherence monitoring to improve scalability and translational impact. The preventive potential of NMT was only realised when high compliance bridged the implementation gap, ensuring the effective translation of biomechanical principles into sustained protection against sex-specific injury disparities.

## Supplementary Material

Appendix A.docx

## Data Availability

The datasets generated and analysed during the current study are available in the supplementary material of this article. Additional de-identified data supporting the findings are available from the corresponding author upon reasonable request.
