# Supplementary material for: Neuromuscular training for preventing knee injuries in female team athletes: a meta-analysis
Source: Ann Med. 2025 Nov 1;57(1):2581891. doi: 10.1080/07853890.2025.2581891 (PMC12581765; doi:10.1080/07853890.2025.2581891)
Supplement: Appendix A.docx [file IANN_A_2581891_SM2515.docx]

Appendix A. Search Strategies for All Databases

Table A1. Detailed database retrieval strategies used in the present meta-analysis.

| **Database** | **Retrieval Strategy** |
| --- | --- |
| **PubMed** | #1 "knee injuries"[MeSH] OR "anterior cruciate ligament injuries"[MeSH] OR "anterior cruciate ligament"[MeSH] OR "knee injury"[tiab] OR "ACL injury"[tiab] OR "ACL injuries"[tiab] OR "lower extremity injur*"[tiab]  #2 "female"[MeSH] OR "female athlete*"[tiab] OR "woman"[tiab] OR "women"[tiab] OR "girl"[tiab] OR "young female*"[tiab] OR "adolescent female*"[tiab]  #3 "neuromuscular training"[tiab] OR "exercise program"[tiab] OR "exercise intervention"[tiab] OR "warm-up"[tiab] OR "injury prevention"[tiab] OR "prevention program"[tiab] OR "injury reduction"[tiab] OR "preventive training"[tiab] OR "strength training"[tiab] OR "balance training"[tiab] OR "plyometric training"[tiab]  #4 #1 AND #2 AND #3 |
| **Web of Science** | TS=("knee injur*" OR "ACL injur*" OR "lower limb injur*" OR "knee trauma" OR "sports injur*" OR "lower extremity injur*" OR "leg injur*") AND ("neuromuscular training" OR "injury prevention" OR "exercise program" OR "training program" OR "exercise intervention" OR "warm-up" OR "physical training") AND ("female" OR "women" OR "girls" OR "female athlete*" OR "female players" OR "adolescent female" OR "young women")  Refined by: Document type = Article |
| **Embase** | #1 'knee injury'/exp OR 'anterior cruciate ligament injury'/exp OR 'acl injury':ti,ab OR 'knee trauma':ti,ab  #2 'female'/exp OR 'female athlete*':ti,ab OR 'women':ti,ab OR 'girls':ti,ab  #3 'neuromuscular training':ti,ab OR 'warm-up':ti,ab OR 'injury prevention':ti,ab  #4 #1 AND #2 AND #3 |
| **Cochrane** | ("anterior cruciate ligament" OR "knee injury") AND ("female athlete" OR "female soccer players" OR "female football players") AND ("neuromuscular training" OR "FIFA" OR "structured warm-up" OR "injury reduce") AND ("RCT" OR "Randomized controlled trial") |
| **Scopus** | (TITLE-ABS-KEY("knee injuries" OR "ACL injuries" OR "lower limb injur*" OR "lower extremity injur*" OR "anterior cruciate ligament injuries" OR "anterior cruciate ligament")) AND (TITLE-ABS-KEY("female" OR "female athlete*" OR "women" OR "girls" OR "young female*" OR "adolescent female*")) AND (TITLE-ABS-KEY("neuromuscular training" OR "neuromuscular exercise" OR "exercise program" OR "exercise intervention" OR "warm-up" OR "warm up" OR "injury prevention" OR "injury reduction" OR "preventive training" OR "strength training" OR "proprioceptive training" OR "balance training" OR "plyometric training")) AND (TITLE-ABS-KEY("team sport*" OR "football" OR "soccer" OR "basketball")) AND (DOCTYPE(ar)) |

Search strategies were conducted from inception to 31 December 2024. Similar strategies were adapted for searches in all databases.
